# Cutaneous squamous cell carcinoma in vertically acquired HIV and epidermodysplasia verruciformis

**DOI:** 10.4102/sajhivmed.v23i1.1368

**Published:** 2022-06-27

**Authors:** Linda A. Mandikiyana Chirimuta, Francis J. Ndowa, Margaret J. Pascoe

**Affiliations:** 1 Newlands Clinic, Ruedi Luethy Foundation, Harare, Zimbabwe; 2 Department of Public Health, Environments and Society, Faculty of Public Health and Policy, London School of Hygiene and Tropical Medicine, Harare, Zimbabwe; 3 Skin and Genito-Urinary Medicine Clinic, Harare, Zimbabwe

**Keywords:** squamous cell carcinoma, HIV infection, epidermodysplasia verruciformis, verruca plana, human papillomavirus

## Abstract

**Introduction:**

Acquired epidermodysplasia verruciformis (EV) is a skin disorder that has been described in individuals with perinatally acquired HIV. Many cases have been identified in sub-Saharan Africa in keeping with the epidemiology of HIV infection compared to the rest of the world, where cases are rare. Epidermodysplasia verruciformis skin lesions may undergo malignant transformation. There are few documented cases of malignant transformation of these skin lesions. We describe a patient with an EV-like skin rash who developed cutaneous squamous cell carcinoma (SCC).

**Patient presentation:**

A 25-year-old man, on antiretroviral treatment for 12 years, presented with a generalised skin rash since the age of 11 years, and a 7-month history of a persistent scalp ulcer. He had no history of trauma, radiation or other chronic conditions. Despite an undetectable HIV viral load, he had failed to immune reconstitute (CD4 42 cells/µL). Physical examination revealed a generalised hypopigmented, papular skin rash resembling verruca plana and a 3 cm × 3 cm ulcer with rolled edges on the right parietal region of the scalp. There were no palpable lymph nodes in the head and neck areas. Biopsy of the ulcer revealed moderately differentiated SCC.

**Management and outcome:**

Wide local excision of the lesion was done under local anaesthesia and histological analysis confirmed completely excised moderately differentiated SCC. Further examination four weeks later revealed two, smaller, histologically similar scalp lesions which were completely excised.

**Conclusion:**

Patients with acquired EV require thorough, frequent examination for skin lesions with possible malignant transformation. Early identification of malignant transformation and treatment with surgical intervention is curative.

## Introduction

Epidermodysplasia verruciformis (EV) is a rare genetic skin disorder, first described by Lewandosky and Lutz in 1992,^1^ and is characterised by disseminated dyschromic, squamous skin macules and flat warts, beginning in childhood and occurring predominantly in sun-exposed areas.^1,2^ Epidermodysplasia verruciformis-like skin lesions have been described in individuals with perinatally acquired HIV and this acquired form of EV is similar, both clinically and histologically, to the autosomal recessive form.^3,4^ Although the prevalence of HIV-associated EV-like skin lesions is unknown, Lowe et al. found EV-like skin lesions in approximately one quarter of hospitalised adolescents in Zimbabwe with perinatally acquired HIV, suggesting that this skin disease is not uncommon in high HIV prevalence settings.^3^

Epidermodysplasia verruciformis skin lesions develop after specific EV-associated human papillomavirus (HPV) types, referred to as beta-HPV, infect individuals at a young age and persist due to genetic or acquired impairment of cell-mediated immunity.^5,6^ In HIV-infected individuals, EV-like skin lesions may be a feature of perinatally acquired HIV infection, rather than horizontally acquired infection.^4^

Epidermodysplasia verruciformis is a pre-malignant condition and non-melanoma skin cancers develop in 30% – 50% of patients with EV.^5,6^ However, only a few cases of malignant change in HIV-associated EV-like skin lesions have been reported.^7^ We describe a patient with extensive EV-like skin lesions who developed multiple foci of cutaneous squamous cell carcinoma (SCC) at 25 years of age.

## Case presentation

A 25-year-old man from Zimbabwe, with vertically acquired HIV infection diagnosed at the age of 12 years, and commenced on antiretroviral treatment (ART). He presented with a scalp ulcer for seven months which had started as a flat wart, became ulcerated and increased in size. There was no history of diabetes mellitus, radiation or trauma to the scalp, and, with the exception of a generalised, non-itchy skin rash for the past 14 years, he had no other chronic conditions.

His baseline CD4 cell count and HIV viral load at the time of ART commencement are unknown. He received zidovudine/lamivudine/nevirapine for ten years before developing virological treatment failure. He was subsequently switched to abacavir/lamivudine/atazanavir/ritonavir, which he was taking at the time of presentation.

**FIGURE 1:**
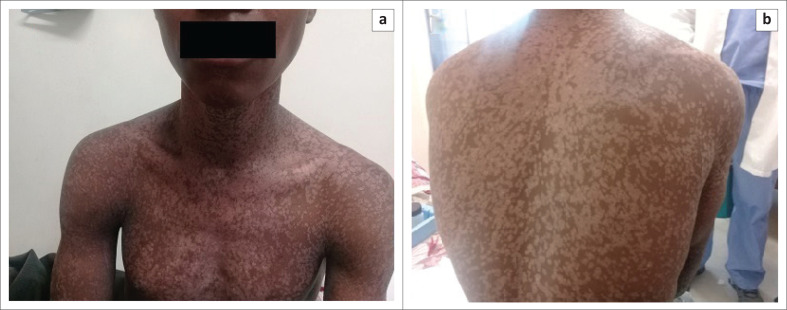
(a and b) Physical examination revealed a generalised hypopigmented macular rash diagnosed as *verruca plana*.

**FIGURE 2:**
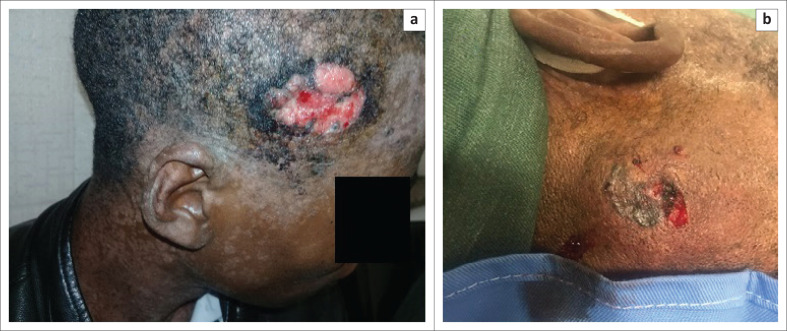
(a) Right parietal scalp ulcer; (b) Left occipital lesion, following a wedge biopsy.

On physical examination he had an extensive hypopigmented, flat-topped, papular rash with the lesions varying from 1 mm to 15 mm in diameter, on his scalp, face, neck, trunk and limbs. The lesions resembled verruca plana (Figure 1). An ulcerating lesion measuring approximately 3 cm × 3 cm in diameter, with rolled edges and an uneven base, was present on the right parietal region of the scalp (Figure 2a). There were no palpable lymph nodes in the head and neck areas, and no oral or genital lesions. Blood investigations revealed severe immune suppression (CD4 cells 42/µL) and an undetectable HIV viral load (< 50 copies/mL).

## Management and outcome

Wide excision of the lesion was performed under local anaethesia, and histology revealed moderately differentiated SCC with the margins reported to be free of tumour.

Repeat examination four weeks later revealed two further hyperpigmented plaque-like scalp lesions, one on the left occipital region, measuring approximately 1.5 cm × 1.5 cm (Figure 2b), and a second on the frontal region, measuring 0.7 cm × 0.7 cm in diameter. Excisional biopsies of these lesions were done and histology revealed completely excised moderately differented SCC with underlying solar keratosis.

## Discussion

We present a patient with perinatally acquired HIV, and acquired EV, whose skin disease progressed while taking ART, and who later developed multifocal SCC.

Epidermodysplasia verruciformis occurs due to infection by oncogenic beta-HPV (including HPV types 3, 5, 8, 9, 10, 12, 14, 15, 17, 19, 25, 36, 38, 47 and 50) in patients with genetic or acquired impaired cell-mediated immunity.^6^ In the genetic form of EV, mutations of EVER1 and EVER2 genes, located on chromosome 17q25, cause down-regulation of cell-mediated immunity, reducing the cell’s ability to present beta-HPV antigens to T-lymphocytes.^8^ Infection with HIV results in destruction of T-helper cells, causing impaired cell-mediated immunity. Beta-HPV types found in inherited EV (mostly HPV types 5 and 8) have also been found in HIV-infected patients with acquired EV.^3,7^ A study in children with HIV infection and acquired EV found that 90% had at least one EV-related HPV type, and over 50% had more than one HPV type detected in skin biopsies.^9^ In addition to being infected with EV-related HPV types, children who have EV-like skin lesions have been found to be co-infected with other HPV strains, including high risk types.^3^ This means they may be at concurrent risk of other HPV-related cancers, particularly anogenital SCC.

The histology of acquired EV lesions is similar to that of inherited EV, with typical blue cells with pallor and mild acanthosis.^3,10^ The EV-like skin lesions in these patients can be recalcitrant to treatments for warts,^9^ are disfiguring and stigmatising, and pose a risk of progression to malignancy. Studies have shown that ART does not have a significant impact on skin disease progression.^3,7^

Malignant transformation has been well documented in patients with inherited EV, but there is a paucity of data regarding malignant transformation in patients who have acquired EV. In 30% – 70% of cases of inherited EV, cutaneous skin cancers develop, and malignant transformation usually develops after 30 years of age.^2,5,6,11^ A case series of patients with inherited EV who developed skin cancers, found that six of the seven cases had cutaneous SCC with multiple foci.^2^ This picture of multiple foci of carcinoma in our patient is similar to the description in this case report. Ultraviolet (UV) radiation acts synergistically with beta-HPV, inducing carcinogenesis by direct damage of DNA and immunomodulatory mechanisms. Therefore, HPV-related skin cancers usually occur in sun-exposed areas.^12^ The patient in this case report resided in a rural area and was probably exposed to prolonged periods of UV radiation during the activities of his daily life.

## Conclusion

We recommend that HIV-infected patients with EV-like skin lesions should undergo regular, thorough physical examination to enable early detection of new lesions and malignant transformation. Because of the risk of other HPV-related malignancies, these patients should undergo regular anogenital examination. Complete surgical excision is curative if lesions are detected prior to metastatic spread. Other preventive strategies include counselling patients on the avoidance of sun exposure, the use of sunscreens, regular self-examination and early reporting of skin changes or abnormal findings.
